# Elevated clozapine levels in patients with COVID-19 infection

**DOI:** 10.1192/j.eurpsy.2021.803

**Published:** 2021-08-13

**Authors:** J. Marti Bonany, E. Pérez Sánchez, M. Pérez Carre, M.I. Martínez Casamitjana, J.R. Fortuny Olive, C. Macias Castellví, E. Carrió Diez, F. Lana Moliner, R. Sánchez González

**Affiliations:** Institut De Neuropsiquiatria I Addiccions, Parc de Salut Mar, Santa Coloma de Gramenet, Spain

**Keywords:** clozapine, COVID-19, levels

## Abstract

**Introduction:**

Clozapine is the most effective antipsychotic for treatment resistant schizophrenia. In patients treated with clozapine, COVID-19 infection may result in complications including an increased risk of pneumonia, clozapine toxicity, and disruption to clozapine treatment by COVID-19 induced lymphopenia.

**Objectives:**

We report 5 cases of elevated clozapine levels occurring in patients with COVID-19 infection who had been previously managed for several years on stable doses.

**Methods:**

Subjects: 48 admitted patients to a long-stay psychiatric unit. COVID-19 infection confirmed by positive nasopharyngeal swab for viral ribonucleic acid of SARS-CoV-2. Hematological controls between March and April 2020.

**Results:**

16 patients (33%) treated with clozapine.18 patients (37’5%) had COVID-19 infection, of which 5 (10’4%) were treated with clozapine. Results are presented in table 1. Increases in plasma clozapine levels were observed in all cases (49’38 to 307.5%). We don’t have the clozapine levels of a patient who presented a pneumonia requiring admission and treatment in the general hospital. Two cases of neutropenia were observed, of which one had to discontinue treatment with clozapine. In the other three patients the dose of clozapine was reduced and they did not present haematological or intoxication complications that required further adjustments.

**Conclusions:**

Covid-19 infection is associated with increased serum clozapine levels by probably multifactorial mechanisms (systemic infection, reduced smoking). Importance of full clinical assessment of suspected COVID-19 infection in clozapine treated patients, including assessment clozapine level, and full blood count. The general recommendation is to reduce the dose of clozapine in this patients.

